# Follow-up and outcome of patients with primary BH4 deficiencies

**DOI:** 10.3389/fneur.2026.1793300

**Published:** 2026-07-16

**Authors:** Francesca Nardecchia, Filippo Manti, Agnese De Giorgi, Serena Galosi, Jennifer Friedman, Vincenzo Leuzzi

**Affiliations:** 1 Department of Human Neuroscience, Sapienza University of Rome, Rome, Italy; 2 Department of Neuroscience/Mental Health, Unit of Child Neurology and Psychiatry, Azienda Ospedaliero Universitaria Policlinico Umberto I of Rome, Rome, Italy; 3 Department of Pediatrics, Division of Genetics, University of California, San Diego, San Diego, CA, United States; 4 Rady Children’s Hospital, San Diego, San Diego, CA, United States; 5 Rady Children’s Institute for Genomic Medicine, San Diego, CA, United States

**Keywords:** BH4 deficiencies follow-up, BH4 deficiencies outcome, Dopa responsive dystonia DRD, dystonia-parkinsonism, movement disorder in children, neurodevelopmental disorders, neurotransmitters synthesis disorders

## Abstract

BH4 deficiencies may occur with or without hyperphenylalaninemia (HPA). If identified early through neonatal screening for PKU, the clinical follow-up aims to prevent the onset of typical disease symptoms. In contrast, for the conditions not associated with HPA, diagnosis usually occurs once symptoms manifest, and follow-up focuses on monitoring the treatment’s effectiveness in restoring normal neurological function. Except for pterin-4-alpha-carbinolamine dehydratase defect (PCCDd), the core symptoms and natural course of these diseases involve neurodevelopmental impairment and movement disorders. Current information on clinical follow-up and outcome comes from retrospective observational studies, with standardized measures used in only a few of them. Serial clinical observations focusing on the two most consistent areas of neurological impairment are the best predictors of outcome and the main follow-up targets. Available clinical data, often aggregating early- and late-treated patients, show that the best clinical outcome occurs in autosomal dominant guanosine triphosphate cyclohydrolase I deficiency (AD-GTPCHd). In contrast, for recessive conditions, prognosis is variably associated with both the timing of treatment (autosomal recessive [AR]-GTPCHd, 6-pyruvoyltetrahydropterin synthase [PTPS]d, sepiapterin [SR]d, q-dihydropyridine reductase [DHPR]d) and the severity of metabolic derangement (AR-GTPCHd, PTPSd). Neurocognitive, psychiatric, and sleep disorders are currently underestimated and can occur in children and adults, affecting social adaptation and quality of life. Among metabolic alterations, blood Phe levels, when altered, warrant regular monitoring. CSF evaluation can be considered in subjects unresponsive to treatment or with an unexpected clinical course. CSF 5-MTHF monitoring should be reserved for patients with DHPRd experiencing neurological deterioration and seizures. Monitoring prolactin levels, when altered, may assist in personalizing pharmacological treatment. Serum magnesium and glucose are metabolic markers that should be assessed in PDCCd. Brain MRI monitoring is recommended for patients experiencing unusual courses, epilepsy, and neurological deterioration (particularly in PTPSd and DHPRd). A DAT scan is advised for those with AD-GTPCH who present or develop Parkinsonism, and EEG monitoring should be conducted for every patient with epilepsy or suspected seizures. For future clinical studies, it is mandatory that the clinical assessment adopt standardized tools that score impairment across neurological and behavioral domains, taking the patient’s age into account.

**Systematic review registration:**

https://www.crd.york.ac.uk/PROSPERO/display_record.php?RecordID=1144143, identifier (CRD420251144143).

## 1 Introduction

**Figure 1:**
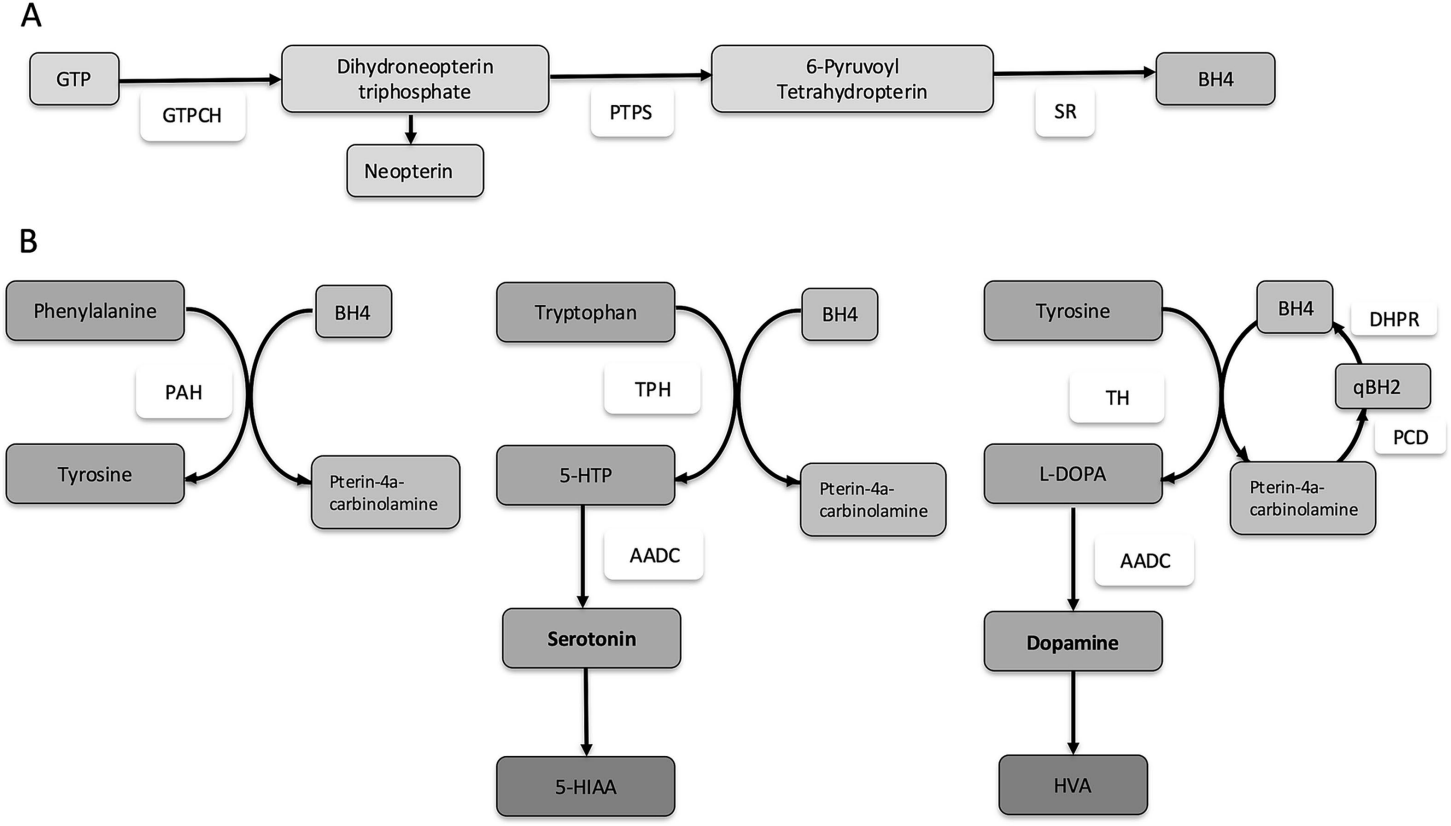
Biosynthesis of BH4 **(A)** and monoamine neurotransmitters **(B)**. AADC, aromatic amino acid decarboxylase; BH4, tetrahydrobiopterin; DHPR, q-dihydropyridine reductase; GTPCH, GTP cyclohydrolase I; HVA, homovanillic acid; 5-HIAA, 5-hydroxyindolacetic acid; PAH, phenylalanine hydroxylase; PCD, Pterin-4-*α*-carbinolamine dehydratase; PTPS, 6-pyruvoultetrahydropterin synthase; qBH2, quinonoid dihydrobiopterin; SR, sepiapterin reductase; TH, tyrosine hydroxylase; TPH, tryptophan hydroxylase.

Tetrahydrobiopterin (BH4) is an essential cofactor for the aromatic amino acid hydroxylases phenylalanine hydroxylase (PAH), tyrosine hydroxylase (TH), and the two isoforms of tryptophan hydroxylase (TPH1 and TPH2) (Figure 1). It also serves as a cofactor for two other enzymes, alkylglycerol monooxygenase (AGMO) and nitric oxide synthase (NOS 1–3) (1, 2).

BH4 deficiencies, among the first genetic causes of movement disorders (MDs) in children for which an effective etiological treatment became available, encompass four conditions associated with hyperphenylalaninemia (HPA) and three not associated with HPA.

The first ones (autosomal recessive guanosine triphosphate cyclohydrolase I defect (AR-GTPCHd), 6-pyruvoyltetrahydropterin synthase defect (PTPSd), pterin-4-alpha-carbinolamine dehydratase defect (PCCDd), and q-dihydropyridine reductase defect (DHPRd)) may be identified through neonatal screening programs for phenylketonuria (PKU). The diagnosis of the second ones (autosomal dominant GTPCH defect (AD-GTPCHd), AR-GTPCHd, and sepiapterin reductase defect (SRd)) follows the traditional clinical approach, based on clinical pattern recognition and related diagnostic testing (1, 2). On clinical grounds, all genetic alterations of the BH4 pathway, except the ultra-rare PCDDd, lead to movement disorders, with or without neurodevelopmental impairment and autonomic dysfunction, depending on the age at onset, which reflects the severity of metabolic failure (3). Follow-up and outcomes clearly differ for conditions diagnosed pre-symptomatically compared with those identified and treated after established neurological impairment. In the first case, clinical follow-up aims to monitor treatment and prevent the emergence of disabling disease symptoms. Conversely, in the second case, the primary goal of treatment and clinical monitoring is to restore impaired neurological functions.

Comprehensive and authoritative overviews (1) and, more recently, guidelines (2) have extensively focused on the biochemical, diagnostic, and treatment aspects, as well as the genetic background (4), of BH4 deficiencies. Nevertheless, despite the long time since their identification and the availability of effective pharmacological treatment, the clinical outcomes of these diseases have been less systematically explored.

In this systematic review, we aim to provide an overview of the available clinical data on the natural history and treatment outcomes of BH4 deficiencies. We also focus on unresolved problems in the clinical monitoring of these diseases and on potential predictive markers of outcomes. Finally, we suggest standardized tools for future clinical studies.

For other aspects of the disease, the reader is referred to the papers mentioned above.

## 2 Materials and methods

**Figure 2:**
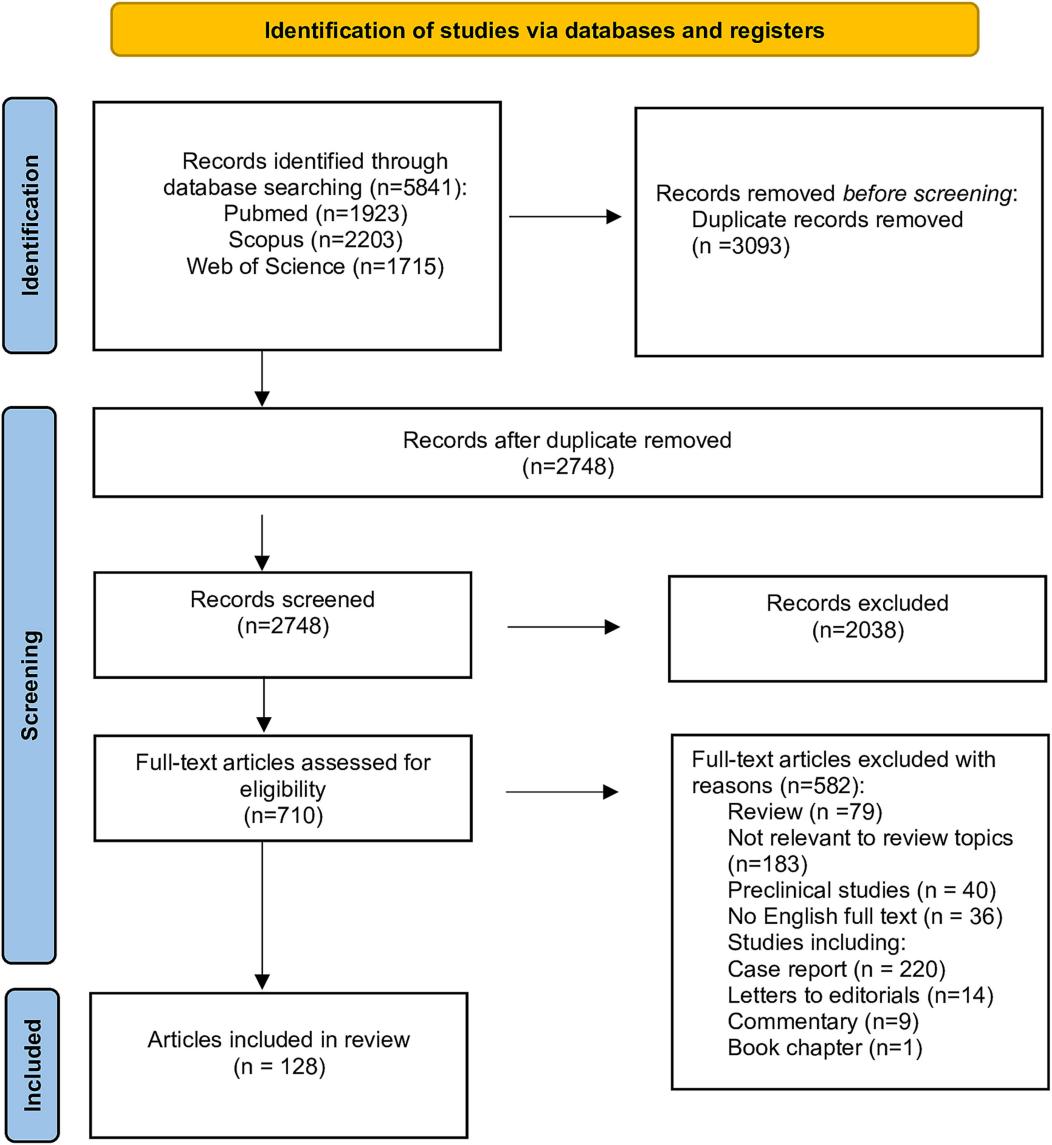
Study flow diagram showing selection of articles for analysis.

A systematic review based on the PRISMA guidelines (PROSPERO code: CRD420251144143), included follow-up and outcome studies on BH4 deficiencies from the first description of the diseases to 31 October 2025. A PubMed, Scopus, and Web of Science search with descriptions of patients with BH4 deficiencies was performed, using the following search terms (“Tetrahydrobiopterin Deficiency” OR “BH4 deficiency” OR “6-pyruvoyl-tetrahydropterin synthase deficiency” OR “PTPS deficiency” OR “dihydropteridine reductase deficiency” OR “DHPR deficiency” OR “sepiapterin reductase deficiency” OR “SRd” OR “pterin-4α-carbinolamine dehydratase” OR “PCBD1” OR “GTP cyclohydrolase I” OR “GTPCH deficiency” OR “GCH1” OR “Guanosine Triphosphate–Cyclohydrolase” OR “Segawa disease” OR “dopa-responsive dystonia” OR “juvenile-onset parkinsonism” OR “cerebral folate deficiency”) AND (“phenotype” OR “outcome” OR “prognosis” OR “follow-up” OR “MRI” OR “metabolic monitoring” OR “biomarkers” OR “prolactin” OR “biogenic amines” OR “parkinsonism” OR “dystonia” OR “movement disorders” OR “phenylketonuria” OR “hyperphenylalaninemia”). The review was conducted in accordance with PRISMA (Preferred Reporting Items for Systematic Reviews and Meta-Analyses) guidelines. The flow of studies through the screening process is presented in the PRISMA flowchart (Figure 2). All English-language papers relevant to the clinical questions were screened and assessed. The review excluded: (a) review articles; (b) preclinical studies; (c) studies not pertinent to the topic; (d) BH4-related studies that were not aligned with the aims of the review or focused on technical aspects without clear clinical implications; (e) case reports; (f) letters to the editor; (g) commentaries; and (h) book chapters. The retrieval and selection of eligible articles followed a three-step process. First, a predefined search strategy was applied to identify relevant publications in the targeted scientific databases. Second, duplicates were removed, and the remaining titles and abstracts were screened; studies that met the initial criteria were then subjected to full-text review according to predefined inclusion and exclusion criteria. Third, data relevant to the study objectives were extracted from the final set of included articles.

All abstracts and full-text articles were independently screened by at least two authors (FM, and AD). Any disagreements were resolved through discussion. Quality assessment of the selected publications was conducted using a quality index adapted from the 9-star Newcastle–Ottawa Scale (NOS). A detailed description of the modified NOS and the assessment procedure is provided in Supplementary Table S1. Data extraction was performed using identical structured forms, which were then compared to ensure consistency and accuracy.

Due to the rarity of the diseases, selected case reports describing atypical phenotypes or distinctive clinical findings relevant to the aims of the review were discussed.

The genetic background of BH4 deficiencies and the relationship between genotype and phenotype, which were the focus of a recent systematic review (4), will not be addressed in the current review. Genetic alterations will be discussed only where current data suggest an association with clinical presentation or outcome.

The ultrarare PCDD deficiency has not been included in this study.

## 3 Results and discussion

The initial database search identified 5,841 potentially relevant articles. After removing duplicates, 2,748 unique manuscripts were screened by title and abstract. Of these, 710 advanced to full-text screening, during which the predefined inclusion and exclusion criteria were applied. Ultimately, 128 studies met all criteria and were included in this systematic review (see Figure 2; Supplementary Table S1).

AD-GTPCHd results will be summarized separately, while recessive forms of BH4 deficiencies, which share a similar neurological phenotype, will be considered together.

### 3.1 Autosomal dominant GTP-CH-I deficiency (AD-GTPCHd)

#### 3.1.1 Symptoms and natural history

The early description of AD-GTPCHd delineated the clinical phenotype and disease course, encompassing the age at onset (childhood to adolescence), a prevalent MD such as dystonic postures of the lower limbs, diurnal fluctuation of motor symptoms (worsening in the evening and improving after sleep), disease progression (a slow generalization of dystonia from lower to upper limbs without the loss of other neurological functions), a self-limiting course (full expression of the disease within 10–15 years from onset), and a dramatic and persistent clinical response to levodopa, irrespective of the patient’s age and disease duration (5).

Sex influences disease penetrance (15% in men, 45% in women), incidence (2.5–23 times higher in females than in males) (6–13), and age at onset (younger in women than in men) (14, 15). Age at onset affects clinical presentation and course: infancy or early childhood onset is associated with motor delay or stagnation that precedes the emergence of dystonia; the classical dopa-responsive dystonia (DRD) phenotype is characterized by onset during childhood or adolescence; after age 15, juvenile or early-onset parkinsonism becomes the prevalent phenotype (6, 7). As a general rule, patients experience a slow, progressive anatomical spread of MD, leading to generalized dystonia, dystonia-parkinsonism, or parkinsonism, depending on the type of MD at presentation. Less common presentations and disease progression have been described, such as neonatal onset (7, 14, 16, 17), spastic paraplegia (18–27), rigid-hypokinetic syndrome (28–30), or myoclonic dystonia (24, 31, 32). Concerning movement disorders, Table 1 summarizes the more and less common patterns of presentation and progression of movement disorders in AD-GTPCHd patients.

**Table 1:** AD-GTPCHd: typical and less common clinical presentations, outcome, and follow-up.

Age at onset	Common symptoms at onset	Disease progression (10–15 years from the onset)	Extended phenotype
Early childhood (<3)	Global or motor developmental delay (15, 62) Reduced growth velocity (slowing of height/length gain) (7)	Asymmetric distal-to-proximal progression of dystonia (N-shaped pattern of spread), with postural tremor extending to the limbs, trunk, cranial, and cervical regions, accompanied by a progressive loss of diurnal fluctuation (5, 7).	Hypotonia (first year of life) (15) Neonatal feeding problems (15, 17) Rigid-hypokinetic syndrome (28–30).
3–10 years	Asymmetric dystonic posturing of the lower limbs (5, 7) Diurnal fluctuation of symptoms (5, 7) Clumsiness of diadochokinetic movements (7) Impaired postural reactions with hyperreflexia and ankle clonus in the absence of a Babinski sign (7)		Oculogyric crisis (7) Progressive spastic paraplegia (19–24, 26, 27) Gait ataxia (20, 28, 54) Dystonic scoliosis (15) Gaze–evoked nystagmus (54) Myoclonic dystonia of limbs and trunk (31) Isolated striatal toe (31) Spontaneous reduction and exacerbation of dystonia (24) Dystonic spasm with or without pain (7) Restlessness (24) Restless leg syndrome (19)
>10 years	Generalized dystonia-parkinsonism (8, 28)	Generalized dystonia-parkinsonism (8, 28)	Adult-onset distal upper-limb rest and action tremor with slow generalization, without dystonia or parkinsonism (58)
Adulthood	Asymmetric parkinsonism (7, 24, 35–37)	Stable levodopa-responsive parkinsonism Impairment of visual and memory-guided voluntary saccades (7, 24, 35–37)	Parkinson disease (41)

Regarding oculomotor function, mild limitation of upgaze and impaired smooth pursuit were reported in AD-GTPCHd patients (32). More recently, the impairment of memory-guided saccade tasks, including increased latency and failure to target, as well as the less effective inhibition and lower latency of reflexive saccades, has been reported and interpreted as the result of the loss of direct basal ganglia pathway inhibitory control on eye movements through the superior colliculus (33). These findings were more prominent in male patients, particularly between 13 and 25 years.

A high rate of relatives with classical Parkinson Disease (PD) without prior DRD has been reported in families with AD-GTPCHd (34.7% of the families in the series by Hagenah et al., 37% by Fernández-Ramos et al.) (28, 34). Moreover, juvenile or early-onset levodopa-responsive parkinsonism has been reported as a late presentation of AD-GTPCHd (6, 23, 35–37). Distinct neuropathological, neurochemical, and neuroimaging features suggest a different background in AD-GTPCHd and PD (6, 25). PD is a progressive disease caused by degeneration of the nigrostriatal pathway, loss of dopaminergic neurons in the ventrolateral region of the substantia nigra (SN), and accumulation of Lewy bodies in the brainstem (nigral cells and locus coeruleus) (38, 39). In contrast, the defect in AD-GTPCHd is a metabolic disorder resulting from dopamine depletion in the nigrostriatal pathway due to secondary tyrosine hydroxylase (TH) deficiency (25). Although neuropathological data are available for only a limited number of DRD cases, they consistently show reduced melanin pigmentation and dopamine content in nigrostriatal neurons, in the absence of nigral neuronal loss or degenerative changes (25).

Nevertheless, the link between parkinsonism and PD related to AD-GTPCHd remains unresolved. The neuropathological features of a patient with DRD associated with the 276delC variant on *GCH1*, who died at 39, exhibited a combination of low melanin content in nigral neurons (as reported in the AD-GTPCHd) and diffuse neuronal loss with reactive gliosis and Lewy bodies in the surviving nigral cells and locus coeruleus (as reported in classical PD) (40). Furthermore, PD relatives of patients with DRD, sharing the same *GCH1* genotype, showed reduced striatal DAT binding values at [123]I-FP-CIT single photon emission computed tomography (SPECT), as detected in typical PD. In contrast, normal values were observed in family members with DRD (29, 41, 42). Finally, whole-exome sequencing of patients with early-onset PD or familial PD (41) revealed a higher occurrence of potentially pathogenic *GCH1* variants than in controls, which was confirmed by a large-scale meta-analysis identifying genome-wide risk loci associated with PD (43). Current data suggest that certain *GCH1* variants may act as risk factors for PD by lowering the threshold for disease development in predisposed individuals or by unmasking subclinical nigral pathology. However, they do not play a primary causal role in nigral neurodegeneration (41, 42).

Nonmotor symptoms: While dopaminergic dysfunction within prefrontal and frontostriatal pathways may contribute to cognitive and behavioral alterations, concomitant serotonergic and noradrenergic deficits have also been implicated in the pathophysiology of nonmotor symptoms in AD-GTPCHd (44). However, the expression of nonmotor symptoms remains highly variable, even among carriers of the same *GCH1* pathogenic variants (7, 45). Although general intellectual abilities are usually preserved (24, 32, 44, 46–50), several reports have documented cases of cognitive impairment in DRD, and dementia has been diagnosed in some individuals with a classical Parkinsonian phenotype (7, 28, 41, 45, 51).

Difficulties in concentration, verbal memory (14, 45) and attention span (52) have also been described in some individuals. Interestingly, Nitschke et al. (53) reported that three patients with DRD exhibited both clinical and neuropsychological improvement following levodopa therapy, while López-Laso et al. (45) observed that all children treated before the age of ten achieved normal cognitive outcomes, with IQ scores within the expected range.

Psychiatric manifestations are more frequent in patients with DRD than in the general population. Reported conditions include major depression, non-reactive mood swings, anxiety disorders, panic attacks, obsessive-compulsive disorder, and eating disorders (11, 14, 44, 51, 52, 54–56). Depressive episodes have been reported to be more frequent before initiation of levodopa therapy, which may help stabilize mood symptoms (44, 46). Psychiatric symptoms also improve with medications that enhance serotonergic and noradrenergic neurotransmission (such as SSRIs, SNRIs, or 5-HTP) (44). In symptomatic individuals, psychiatric symptoms generally do not precede the onset of motor manifestations (14). Their occurrence seems largely independent of motor severity and has also been reported in asymptomatic *GCH1* variant carriers, suggesting that psychiatric comorbidity may represent an intrinsic component of the disease phenotype rather than a secondary psychological reaction to motor impairment (55). In contrast, in the cohort reported by López-Laso et al. depressive and obsessive-compulsive symptoms were absent, anxiety was uncommon, and impulsivity was the most consistent behavioral feature in adult patients (45).

Evidence on sleep disturbances in DRD remains limited and somewhat inconsistent (57). Alves Júnior et al. reported that sleep quality was largely preserved among *GCH1* variant carriers, although many subjects reported mild daytime sleepiness and suboptimal sleep (46). In contrast, some authors documented chronic sleep insufficiency, excessive sleepiness, restless-leg syndrome, and frequent disturbing nightmares, hypothesizing that altered serotonin/dopamine neurotransmission and increased nocturnal body movements, typical of the action-dystonia phenotype, could contribute to these symptoms (11, 44, 45, 56, 58). Consistent with this, Timmers et al. found excessive daytime sleepiness in 73% of patients compared with 24% of controls, along with higher fatigue scores. Both daytime sleepiness and fatigue were associated with depressive symptoms and motor severity (55).

Comorbid deafness was identified in four of eleven subjects from the same family (57). Vitiligo has been reported in two unrelated families (59). Hyposmia was described in a single patient (60). Migraine was similarly observed in four affected members of one family (56). Moderate to severe pain (11, 60, 61) and fatigue (11) have also been described and may reflect a reduced sensory input threshold and dysfunction of frontal and subcortical circuits, with possible further involvement of the corticospinal tract. Comorbid conditions such as insomnia may further contribute to these symptoms.

#### 3.1.2 Clinical follow-up

AD-GTPCHd is a treatable condition. Once diagnosed, clinical follow-up focuses on monitoring treatment response, which informs drug choice and dosage. Controlled studies of AD-GTPCHd treatment have not yet been conducted, and disease-specific, standardized clinical tools to assess the severity of neurological impairment and track treatment-related changes have not yet been developed. Well-established symptom-specific scales are integral to current clinical assessments in neurology. Among others, the Burke-Fahn-Marsden Dystonia Rating Scale (BFMDRS) and the MDS-UPDRS for Parkinson’s Disease are widely used to assess the severity of dystonia and Parkinsonism, respectively, as well as the disabilities arising from these disorders (51, 55, 62–64). These scales assist in monitoring the clinical course during treatment. Clinical follow-up should routinely encompass evaluation for psychiatric and behavioral disturbances, as well as sleep disorders (2).

#### 3.1.3 Monitoring of metabolic alterations

The biochemical diagnostic markers of AD-GTPCHd (pterins in urine and pterins and biogenic amine metabolites in CSF, along with delayed clearance of blood Phe) (65) are not helpful for the clinical follow-up of patients (2). Indeed, no metabolic biomarkers surpass the reliability of clinical observation in evaluating treatment outcomes. However, if the clinical response to the treatment is unsatisfactory, which is a rare event in AD-GTPCHd, a CSF examination of biogenic amine metabolites (5-hydroxyindoleacetic acid [5-HIAA] and homovanillic acid [HVA]) may be considered, especially if the pre-treatment CSF examination had shown a lower-than-normal level of one or both metabolites (2).

#### 3.1.4 Neuroimaging monitoring

Neuroimaging of the presynaptic dopamine (DA) system is crucial for differentiating levodopa-responsive Parkinsonism from neurodegenerative PD, particularly when Parkinsonism presents as either an initial or late symptom, which often occurs in adulthood (41). The progressive loss of DA neurons in PD can be assessed *in vivo* using two radiolabeled tracers: [F-18]FDOPA positron emission tomography (PET) imaging primarily reflects striatal dopa decarboxylase activity, whereas [123]I-FP-CIT SPECT evaluates the striatal density of DA reuptake transporter (DAT) (66, 67). Generally, both tracers measure the decline in DA neuron density in the striatum, if present. [123]I-FP-CIT SPECT signals correlate more closely with the remaining number of DA neurons in both early and advanced-stage models of PD (68). Neuroimaging findings are typically normal in individuals with DRD or other forms of levodopa-responsive non-degenerative parkinsonism (2, 27, 47, 48, 50, 51, 56, 69–82). However, several reports have described *GCH1* variant carriers with reduced DAT binding (41, 47, 83–86). These patients typically present with adult-onset dystonia-parkinsonism or isolated parkinsonism without dystonia, often with a slowly progressive course and excellent or sustained levodopa responsiveness. Among *GCH1* variant carriers with reduced DAT binding, only two exceptional cases have been documented: one patient with juvenile DRD (47) and one asymptomatic subject (86). In these instances, although some authors have considered the coexistence of two disorders, a phenotypic continuum has also been proposed (83–85), with chronic DA deficiency potentially predisposing to nigral neurodegeneration (41). In AD-GTPCHd patients presenting with parkinsonism, imaging studies should be considered when there is evidence of clinical progression or a waning levodopa response. Kostić et al. investigated 9 genetically confirmed *GCH1*-related DRD patients, who typically presented with childhood or adolescent onset, very long disease duration, normal [123]I-FP-CIT SPECT findings, and only mild residual motor symptoms. Their MRI evaluation revealed cortical thinning in sensorimotor and associative regions, together with increased volumes of the putamen and pallidum, changes thought to reflect dystonia-related compensatory remodeling rather than neurodegeneration. Diffusion-tensor imaging additionally demonstrated predominantly right-sided white-matter microstructural abnormalities affecting motor, associative, and interhemispheric pathways. Overall, the findings suggest subtle but widespread grey- and white-matter alterations in *GCH1*-related dystonia, indicating disrupted sensorimotor network organization beyond the traditionally assumed purely neurochemical deficit (87).

#### 3.1.5 Neurophysiological examination

Using paired-pulse transcranial magnetic stimulation, Hanajima et al. demonstrated that short-interval intracortical inhibition is preserved in patients with Segawa disease, in contrast to focal dystonias; this finding indicates that motor cortical GABAa-mediated inhibitory circuits are functionally intact and supports the view that dystonia in Segawa disease arises from a reversible dysfunction of basal ganglia–cortical signaling rather than from primary cortical disinhibition, helping to explain its distinct clinical course and favorable long-term outcome (88). More recently, a single-center study using a comprehensive transcranial magnetic stimulation–based neurophysiological assessment—including corticospinal input–output curves, short-interval intracortical inhibition, and paired associative stimulation—found that patients with AD-GTPCHd exhibit largely preserved finger-tapping kinematics, consistent with minimal or absent bradykinesia at the time of evaluation. Despite this relatively spared motor performance, transcranial magnetic stimulation consistently revealed abnormalities of motor cortex physiology, including flattening of the corticospinal input–output curve, reduced intracortical inhibition, and attenuated PAS-induced plasticity in most patients, indicating that cortical excitability and plasticity changes may persist even when clinical motor impairment is mild. This pattern is consistent with clinical reports showing that parkinsonian signs in this condition are often mild and can be favorably influenced by treatment (89).

#### 3.1.6 Outcome

The response to dopaminergic treatment is generally excellent to satisfactory and remains stable in most patients with AD-GTPCHd treated with levodopa (8–13, 15, 17, 24, 27, 32, 47–52, 56, 60–63, 69, 77–80, 82, 88, 90–102). Over time, the required dose of levodopa generally does not need to be increased; in fact, it may even be reduced, reflecting the non-degenerative nature of the disorder (69). In some patients with *GCH1*-related dystonia and parkinsonism, long-lasting remission has been reported, with symptoms disappearing for years, even after treatment discontinuation, and re-emerging later in life. Upon reintroduction, responsiveness to levodopa is typically restored (17, 93, 95).

Longitudinal neurophysiological studies have shown no evidence of clinical or functional progression, with sustained levodopa responsiveness associated with preserved cortical excitability and motor network integrity over follow-up periods of up to 5 years. These findings support the view that AD-GTPCHd represents a functional rather than degenerative dopaminergic disorder (62).

Residual motor signs, such as dystonia or Parkinsonism, affect approximately 15–30% of the treated patients (8, 11, 49, 52, 56, 60, 61, 63, 77, 78, 91, 102, 103), with men experiencing residual impairment more frequently (47.1% of those with dystonia and 19.5% with Parkinsonism) than women (25.6 and 13.9%, respectively) (63). Patients with early onset (<6 years) are more severely disabled than those with later onset (63). In the cohort described by Fernández-Ramos et al. (28), among 53 patients, lack of responsiveness to levodopa was observed in 5 out of 16 patients with tremor, 7 out of 35 with dystonia, and 1 out of 2 with parkinsonism. Factors such as sex, clinical phenotype, diagnostic latency, and age did not predict treatment responsiveness. However, a gait analysis study suggested that earlier treatment initiation may still influence specific locomotor outcomes, particularly gait pattern and foot positioning during walking, with additional benefits in walking speed and overall gait pattern in some patients. Conversely, delayed diagnosis or previous unnecessary orthopedic surgery may contribute to persistent gait abnormalities despite adequate therapy (104).

Using pre-movement somatosensory evoked potential gating, Kimura et al. proposed that Segawa disease comprises two clinically distinct phenotypes: an early-onset postural dystonia with preserved sensorimotor integration and sustained responsiveness to levodopa, and a later-onset action dystonia with additional dystonic movements, reduced levodopa responsiveness, and impaired basal ganglia–thalamo–cortical integration (100).

Regarding treatment tolerance, levodopa-induced dyskinesias in *GCH1* deficiency were reported by several papers and are usually mild, non-progressive, and tend to resolve after dose reduction (15, 28, 47, 49, 51, 61, 91, 105). An unusual case of long-lasting dyskinesias was reported in a child with *GCH1* deficiency, in whom choreiform movements persisted for several years after levodopa initiation but eventually resolved spontaneously despite continued therapy (48). Wearing-off phenomena are rarely observed, even after years of treatment (28). Although some patients reported a subjective sense of wearing-off after missing a dose, objective testing showed a long-duration response with no motor worsening for over 24 h, suggesting that perceived fluctuations were non-motor rather than true motor phenomena (82). Rudakou et al., described a patient who developed Parkinson’s disease at age 48, initially responding to levodopa but later developing dyskinesias and on/off fluctuations, along with anxiety, hallucinations, and probable REM sleep behavior disorder; due to these motor complications, he ultimately underwent successful deep brain stimulation (DBS) (96). In rare patients, levodopa treatment was poorly tolerated or ineffective, whereas anticholinergic therapy could improve dystonia (24, 32). Daida et al. reported two affected women—one presenting with DRD and one with Parkinsonism—who, after several years of levodopa-responsiveness, developed wearing-off phenomena and troublesome dyskinesias. [123]I-FP-CIT SPECT demonstrated presynaptic dopaminergic dysfunction. Bilateral subthalamic nucleus (STN) DBS led to marked and sustained improvement in motor fluctuations and dyskinesia, allowing a significant reduction in dopaminergic medication (64). Furthermore, a child presenting with infantile severe hypotonia and psychomotor delay experienced only partial improvement with levodopa treatment, with motor and developmental disability persisting. Two relatives carrying the same heterozygous *GCH1* variant remained entirely asymptomatic (106). A dystonic crisis induced by aspartame was reported in a male with early-onset DRD (107). Aspartame serves as a source of Phe, and even a mild increase in this amino acid may further reduce tyrosine hydroxylase activity, which is already diminished due to BH4 deficiency.

Pregnancy: AD-GTPCHd is compatible with a normal pregnancy and delivery. No increased frequency of spontaneous abortions has been reported in large families with multiple affected women. Continuing or adjusting dopaminergic treatment prevents neurological symptoms, and there is no evidence that it adversely affects pregnancy, delivery, or the fetus (108–110).

### 3.2 Autosomal recessive disorders of BH4 metabolism

#### 3.2.1 Symptoms and natural history

Despite differences among conditions and within-disease variability, the four autosomal recessive disorders (AR-GTPCHd, PTPSd, SRd, DHPRd) share a similar phenotype spectrum that usually presents in infancy or early childhood. All are characterized at onset by: (1) neurological and neurodevelopmental stagnation and/or regression, with greater severity associated with earlier ages of onset; (2) motor disorders including hypotonia, hypotonic or rigid hypokinesia, tremors, and oculogyric crises; (3) autonomic and behavioral disorders (2, 111, 112) (see Table 2). Delays in treatment lead to neurological and neurocognitive impairments that may be less responsive (MD) or significantly less responsive (cognitive impairment) to therapy (111). Rarer and milder late presentations, typically manifesting as DRD or dystonia-parkinsonism, have been reported for SRd (113–115), PTPSd (116, 117) and AR-GTPCHd (118). Metabolic biomarkers that help differentiate between conditions include blood HPA, pterin alteration patterns in urine and CSF, and monoamine catabolites in CSF (2).

**Table 2:** Autosomal recessive BH4 disorders (AR-GTPCHd, PTPSd, SRd, DHPRd): common clinical presentations and natural history.

Phenotypes	AR GTPCH I deficiency *GCH1*		PTPS deficiency *PTS*		SR deficiency *SPR*	DHPR deficiency *QDPR*
	with HPA	without HPA	Severe form	Mild form	–	–
Blood Phe (NBS)	↑↑ Elevated	Normal	↑↑ Elevated	↑↑ Elevated	Normal	↑↑ Elevated
Age of onset	Neonatal/early infancy (118, 119)	Infancy/early childhood (118, 119)	Neonatal/early infancy (111, 112)	Neonatal/early infancy (111, 112)	Infancy/rare late onset cases (~5–6 years) (2, 115)	Neonatal/early infancy (2, 111)
Common symptoms at onset.	Early-onset hypotonia, hypokinesia, tremor, oculogyric crises; possible late-onset dystonia-parkinsonism.	DRD, diurnal fluctuation, gait disorder.	Hypotonia, oculogyric crises, dystonia-parkinsonism.	Slight developmental delay (or even normal early development).	Oculogyric crises, tremor, developmental delay, severe dystonia-parkinsonism, limb spasticity.	Hypotonia, epilepsy, DEE, dystonia-parkinsonism.
Disease progression.	Intellectual disability; dystonia-parkinsonism.	Neurodevelopmental stagnation and late-onset dystonia-parkinsonism DRD.	Intellectual disability; dystonia-parkinsonism.	No further progression.	Intellectual disability; dystonia-parkinsonism.	Intellectual disability; dystonia-parkinsonism, DEE.

DDE, Developmental and epileptic encephalopathy; DRD, Dopa-responsive dystonia.

PTPSd, DHPRd, and approximately 50% of patients with AR-GTPCHd with HPA (118) can be identified pre-symptomatically through neonatal screening and benefit from early treatment (119). The prognosis is worse for patients diagnosed while already symptomatic. However, even with early treatment, patients with the DHPRd face a higher risk of severe epilepsy and neurological deterioration (111, 120–125). Late-treated patients are at a higher risk of developing epilepsy compared to those who received early treatment (124). Nevertheless, severe drug-resistant epilepsy has been reported in DHPRd patients who were treated early and maintained good metabolic control, possibly due to Central Folate Deficiency (CFD) (124, 125). A few patients with SRd (115) and AR-GTPCHd (118, 126) exhibited epilepsy during infancy and early childhood before treatment began. Among adults with PTPSd, epilepsy persists only rarely after treatment initiation (127). Mortality was higher in patients with DHPRd, followed by those with PTPSd, and in those late or not treated. Fewer deaths were reported for AR-GTPCHd, and none for SRd or PCDDd (111). However, these two conditions are rarer than the above and probably underdiagnosed.

Postnatal somatic growth has not been systematically explored in patients with recessive defects of BH4 synthesis. Dopamine regulates growth hormone (GH) and thyroid-stimulating hormone (TSH) release through hypothalamic D2 receptor activation, potentially impacting normal growth when diminished. Furthermore, both GH and peripheral norepinephrine deficiencies may increase the risk of hypoglycemic episodes (128, 129).

Genetic background is associated with milder phenotypes in patients with PTPSd and DHPRd. Mild phenotype in PTPSd has been associated with variants c.46C>T/p.Arg16Cys, c.78G>T/p.Leu26Phe, c.338A>G/p.Tyr113Cys, and c.370G>T/p.Val124Leu on PTS. Concerning *QDPR*, variants c.451G>A/p.Gly151Ser and c.635T>G/p.Phe212Cys, and c.199-1G>T have been associated with less severe clinical presentations (4). For AR-GTPCHd, 14 heterozygous or homozygous variants were associated with the most severe clinical presentation and course (hyperphenylalaninemia) (118).

#### 3.2.2 Clinical follow-up

Follow-up for autosomal recessive BH4 deficiencies focuses on monitoring the response to treatment of neurodevelopmental and MD, metabolic alterations, and, in some cases, neurophysiological and neuroimaging alterations when they occur. Current clinical knowledge about the outcome primarily relies on retrospective observational studies (111, 115, 116, 123, 130, 131). Recently, an international collaborative effort led to a cross-sectional retrospective study that used standardized metrics of IQ, Quality of Live (QOL), and a self-report questionnaire addressing behavioral issues from patients in the iNTD register (132). The survey included both early- and late-treated patients, as well as several other metabolic neurotransmitter disorders. Developmental quotients (DQ) and IQ are the most used measures of cognitive outcomes (132). Despite the well-established role of dopamine in the development of higher cognitive functions, particularly executive functions (EF), and the influence of these functions on adaptive behavior (AB), only a few papers have included standardized tools to assess both EF and AB in young adult patients (117, 127). The occurrence of psychiatric disorders has only recently become the focus of systematic observation (127, 132, 133). The high risk of intellectual disability and psychiatric disturbances, even in early-treated patients, emphasizes the need for careful monitoring of these individuals using standardized tools. Systematic and standardized tools are also largely lacking in studies on AR-GTPCHd (118), SRd (115), PTPSd (116, 127), and DHPRd (123). An adapted Unified Parkinson’s Disease Rating Scale (UPDRS) has been used to evaluate the effects of non-ergot dopamine-mimetic drugs in small cohorts of adult patients with PTPSd (134) and DHPRd (135).

Nonmotor symptoms: Sleep disorders are a common issue in BH4 deficient subjects as consequence of serotonin depletion. They occur most frequently in SRd (2, 115).

Somatic growth deserves to be closely monitored in children with BH4 deficiencies. If growth is delayed or plateaus, defects in GH and TSH should be considered (129).

#### 3.2.3 Monitoring of metabolic alterations

The key metabolic markers for diagnosing BH4 deficiencies include blood phenylalanine (Phe) concentration, pterin excretion patterns in urine, pterins and biogenic amine metabolites in cerebrospinal fluid (CSF). Other potentially relevant markers are CSF 5-methyltetrahydrofolate (5-MTHF) and blood prolactin. Monitoring blood Phe levels is crucial for patients with HPA. In patients with AR-GTPCHd and PTPSd, BH4 supplementation can normalize blood Phe levels, which is a priority for those with epilepsy (PTPSd and DHPRd) (2).

Regarding biogenic amines in CSF, the pre-treatment levels of HVA and 5-HIAA are within the normal range in approximately one-third of patients with AR-GTPCHd (118) and PTPSd (116), a few with DHPRd (123, 136–138), and virtually none with SRd (115). Normal values or mild depletion of biogenic amines in the CSF are associated with mild clinical presentations and better outcomes for movement disorders and cognitive impairment (116, 118, 127). The value of serial CSF neurotransmitter assessments for optimizing therapeutic replenishment of brain neurotransmitters has not been systematically studied. Retrospective analysis of 121 CSF examinations from 28 patients with PTPSd under treatment showed that levels of 5-HIAA and HVA were below the reference range in 45.5 and 24.0% of cases, respectively. Moreover, among patients with restored normal value, regardless of treatment, 5-HIAA and HVA decline over time to below the reference range in 37.3 and 10.2% of samples, respectively. This decline may lead in some cases to a late emergence of movement disorders in a previously asymptomatic patient. CSF data from 13 patients who underwent at least 4 samples during the entire follow-up suggest a higher probability of movement or psychiatric disorders in subjects with 5-HIAA and HVA below the normal range. Meanwhile, intellectual outcomes do not appear to correlate with the degree of restoration of both CSF 5-HIAA and HVA (127).

In conclusion, the serial assessment of neurotransmitter metabolites in CSF should not be part of the routine follow-up for patients with early-treated BH4 deficiencies, as it does not surpass the value of clinical observation for therapeutic decision-making. The presence of symptomatic patients despite normal levels of biogenic amines in CSF indicates an inconsistent relationship between CSF and intraneuronal levels of neurotransmitters, contrasting with findings in other biogenic amine synthesis disorders (127, 139). However, for patients who are unresponsive, or exhibit less-than-expected responsiveness, to dopaminergic/serotoninergic treatment, or show a divergent response (for example, improvement in movement disorders but persistent cognitive impairment or sleep disorder), or face delayed somatic developmental growth, CSF examination can aid in personalizing pharmacological treatment.

Among BH4 deficiencies, DHPRd is at risk of developing CFD, as one of the metabolic consequences of the disease. 5-MTHF is essential for CNS function, as it plays a role in the synthesis and remethylation of methionine, thereby maintaining a stable production of S-adenosyl methionine (SAM), the primary methylating agent in cells. CFD due to genetic disorders of 5-MTHF synthesis or transport in the brain leads to early-onset severe encephalopathy with epilepsy, white matter alterations, and brain calcification (140, 141). In early-treated DHPRd patients, depletion may occur late and insidiously despite normal peripheral folate levels. The pathogenesis of CFD in the DHPR defect is linked to low brain dihydrofolate reductase (DHFR) expression and a shift in DHFR toward BH4 rather than THF synthesis, leading to brain 5-MTHF depletion (142, 143).

Despite the lack of a systematic analysis of CSF 5-MTHF in relation to clinical outcomes, the most severe clinical course of patients with DHPRd, along with a higher incidence of severe epilepsy (123), suggests that CFD may contribute to the disease’s outcome in some patients. Irons et al. (142) report on a boy who experienced marked neurological deterioration after discontinuing folinic acid and a rapid recovery after treatment was reintroduced. A sudden epileptic status followed by a stroke, with multifocal cortical and subcortical brain alterations accompanying a rapid decline of CSF 5-MTHF, has been reported in a previously normal 10-year-old girl despite the excellent life-long metabolic control (125). A second girl developed generalized epileptic seizures and cerebral and cerebellar edema at age 12 after the interruption of folinic acid supplementation. Prompt folinic acid resumption achieved complete seizure control and improved neuroimaging alterations (124). An acute encephalopathy resembling CFD was reported in an early-treated boy at age 12 who had not received folinic acid supplementation (144).

Waiting for more systematic studies, CSF monitoring of 5-MTHF should be suggested for DHPRd patients who develop epilepsy or clinical signs of neurological deterioration.

The failure or delay to thrive should suggest ruling out central growth-hormone and thyroid hormones deficiency hypothyroidism by testing GH release in preventing spontaneous hypoglycemia occurring after overnight fasting, serum dosage of insulin-like growth factor 1(IGF-1), insulin-like growth factor binding-protein (IGF-BP3), TSH, T3 total triiodothyronine, fT3 free triiodothyronine, T4 total thyroxine, fT4 free thyroxine (129).

Prolactin: The tuberoinfundibular dopaminergic pathway inhibits prolactin secretion. When elevated, as in BH4 deficiency, prolactin reflects the severity of brain dopaminergic depletion and may serve as a central marker of dopamine repletion and restoration. Fluctuations in blood prolactin mirror the dynamics of the dopaminergic effect on the tuberoinfundibular pathway (134, 135). Non-ergot dopamine agonists tend to normalize prolactin levels in PTPSd and DHPRd (134, 135, 145). They may be effective even in patients with a BH4 deficiencies experiencing hyperprolactinemia unresponsive to levodopa treatment (145, 146). When altered, prolactin may serve as a brain marker for drug dosage tuning and treatment adherence (135, 147–150).

#### 3.2.4 Neuroimaging monitoring

Brain MRI monitoring is not indicated for early-treated, asymptomatic patients with AR-GTPCHd, PTPSd, and SRd. In symptomatic cases, an MRI examination is often performed as part of the diagnostic workup before a diagnosis is made. The decision to perform additional MRI examinations depends on clinical conditions and detected alterations (2). Regardless of the disease, the appearance of new symptoms, such as epilepsy or neurological deterioration, despite treatment, should prompt consideration of brain MRI examinations. This is especially relevant for patients with DHPRd. Across mixed published cohorts, brain MRI abnormalities have been reported in approximately 45–65% of patients with BH4 deficiency, most commonly delayed myelination and T2/FLAIR white-matter hyperintensities, together with cortical atrophy, corpus callosum hypoplasia, and occasional basal ganglia or cerebellar calcifications, the latter particularly noted in DHPRd (127, 151–154).

Overall, neuroimaging findings in PTPSd show marked heterogeneity, ranging from delayed myelination and white-matter abnormalities to entirely normal MRI scans, with no consistent correlation to neurological severity. One of the most detailed case series reported delayed myelination at diagnosis in eleven affected patients, including both early- and late-treated individuals (9 and 2 respectively). The abnormalities most consistently involved the frontal lobes, followed by the occipital lobes, the corpus callosum, the temporal lobes, the parietal lobes, and, less frequently, the cerebellum (1 patient). On follow-up imaging, these alterations showed a progressive trend toward normalization, with only minor residual changes persisting and no clear correlation with clinical outcomes (155).

In contrast, several cohorts reported predominantly normal MRI findings. In a group of older PTPSd patients, MRI was normal in 12 of 18 cases. Likewise, in a cohort of 19 newborn-screened patients, imaging was largely unremarkable. In these latter two series, when present, the most common alteration was a stable periventricular white-matter hyperintensities (127, 156). Chien et al. (157) detected structural MRI changes in just one of eight early-treated patients. However, MR spectroscopy identified metabolic alterations, including lactate peaks and reduced N-acetylaspartate ratios, suggesting subclinical neuronal injury despite normal MRI appearances. A transient reduction in Apparent Diffusion Coefficient values within the central tegmental area was also reported in an asymptomatic two-month-old infant receiving early treatment (158). In a Filipino series, MRI was performed in 4 patients: 2 showed focal posterior-lobe atrophy, whereas the remaining 2 had normal scans; importantly, radiologic findings did not correlate with clinical or biochemical profiles (159). Intracerebral calcifications are rare in PTPSd patients (155).

Neuroimaging findings in DHPRd are more heterogeneous than in other BH4 disorders, likely reflecting the combined effects of HPA and secondary cerebral folate deficiency (CFD).

Cortical atrophy was detected by CT imaging in 3 of 4 patients (142). MRI imaging showed white- and gray-matter abnormalities, including supratentorial white-matter, cortical and cerebellar gray-matter, substantia nigra, and incomplete hippocampal inversion, in eight of nine patients (160). Posterior white-matter T2 hyperintensities, cerebral atrophy, and multiple small porencephalic cysts were described in a patient with refractory Lennox–Gastaut–type epilepsy, movement disorders, and profound developmental derangement (161). Regarding the possible consequences of CFD, acute hyperintense cortical and subcortical alterations were identified in the two aforementioned girls, leading to a severe multifocal stroke in one case (125) and improvement with prompt folate supplementation in the other (124). Diffuse signal alteration of the deep white matter occurred in another patient with a similar clinical course, where CFD was not tested (143). Intracerebral calcifications are reported in late-treated subjects (111, 120–122, 142, 162, 163). In contrast to the above-reported cases, normal brain MRI scans were found in four DHPRd patients, including both early-treated asymptomatic infants and late-diagnosed clinically affected individuals (164). No consistent neuroimaging alterations have been found in AR-GTPCHd or SRd (2, 115).

#### 3.2.5 Neurophysiological examination

Electroencephalography (EEG) monitoring is part of the clinical follow-up of all patients with BH4 deficiencies who experience epileptic seizures. Nonspecific EEG abnormalities were reported in 87.5% of patients with BH4 deficiency in the series by Bozaci et al. (151). EEG recording protocols and criteria depend on the type and severity of the seizures, according to the international guidelines for the treatment and monitoring of epilepsy. Pathological EEG patterns were recorded in 89% of DHPRd patients and 79% of PTPSd patients, respectively, which are the two conditions most frequently associated with epilepsy (111, 165). In the study by Ray et al., three patients with DHPRd showed multifocal interictal epileptiform discharges, hypsarrhythmia, or electrical status epilepticus during slow-wave sleep. In another patient, generalized 1–2 Hz slow spike–wave discharges during both wakefulness and sleep coincided with severe developmental impairment and refractory seizures (152).

EEG abnormalities were mild or absent in a cohort of 19 early-treated patients with PTPSd. When present, they consisted of diffuse background slowing without epileptiform discharges. Seizures, when present, were infrequent and easily controlled with standard antiepileptic therapy (156).

A clinical and neurophysiological study comparing patients with dominant and recessive BH4 deficiencies found that motor impairment was more severe in patients with recessive forms, as reflected in bradykinesia, reduced movement amplitude, and irregular movement rhythm, with no sequence effect during repetitive movements, as seen in advanced Parkinsonian syndromes or atypical Parkinsonisms. Prominent abnormalities of motor cortical physiology, including reduced short-interval intracortical inhibition and alterations of the corticospinal input–output curve, have been reported in Parkinson’s disease (89).

#### 3.2.6 Outcome

Information on the outcomes of recessive BH4 deficiencies is primarily based on retrospective observational studies that often include patients treated both early and late. Neurological outcomes vary according to the specific enzymatic defect, the timing of diagnosis, and treatment compliance (151–154, 166, 167). Generally, the earlier treatment is initiated relative to symptom onset (BH4 deficiencies without HPA) or confirmation of a neonatal screening diagnosis (BH4 deficiencies with HPA), the better the outcome (111). However, for certain disorders, such as AR-GTPCHd and PTPSd, the severity of biochemical impairment affects clinical outcome (116, 118). Furthermore, unlike in PKU, early treatment does not prevent neurological impairment in all patients, and a significant number of patients become symptomatic despite treatment (111, 151, 168). A recent survey of outcomes for 86 BH4-deficient participants, including both early- and late-treated patients, revealed that, aside from the normal IQ observed in patients with AD-GTPCHd, significant variability was detected across all those with recessive disorders. A negative correlation was found between age at diagnosis and IQ among individuals with PTPSd. Nevertheless, low IQ values were also seen in PTPSd patients diagnosed early in life. Regarding behavioral disorders, dysregulation of food intake (AR-GTPCHd), phobic fears (PTPSd), and sleep difficulties (SRd) were reported. Finally, regarding quality of life, individuals with BH4 deficiencies reported significantly higher scores than those with other neurotransmitter disorders, along with a higher mean IQ (132).

According to the recent work by Novelli et al. (118)—who reviewed all published cases and added four newly described patients for a total of 45 individuals—the most severe biochemical phenotype of AR-GTPCHd, often associated with HPA, can lead to substantial neurological damage when treatment is delayed. Within this group, 24 patients were classified as early-infantile encephalopathy. Among them, one third developed permanent neurodevelopmental impairment, including global developmental delay and intellectual disability, while others (about 40%) evolved toward a disabling generalized dyskinetic or spastic-dyskinetic cerebral palsy.

In the subgroup of seven patients with a moderate metabolic defect, the most favorable outcomes, seen in those who began treatment within 14 months of symptom onset, ranged from complete clinical normalization to sustained improvement with or without residual focal movement abnormalities. By contrast, more delayed therapy was associated with poor responsiveness and the progression to severe, generalized dystonia or spasticity, often complicated by limb deformities and dysarthria. However, cognitive abilities remained relatively preserved in some cases.

Finally, the few individuals with the mildest metabolic alteration presented with features overlapping with the autosomal dominant form and, like that, responded well to treatment (118). Several cross-sectional, retrospective studies have investigated clinical outcomes in short cohorts of PTPSd patients. Clinical response varied with age at diagnosis and the timing of treatment initiation: patients with delayed diagnosis and late treatment initiation showed persistent neurological impairment (126, 161, 165, 169–173). Nevertheless, long-term follow-up demonstrated a gradual, sustained improvement in both motor and intellectual abilities under chronic supplementation with BH4 and neurotransmitter precursors. In the cohort of late-treated patients described by Lee et al., those with the most severe forms exhibited a mean increase in full-scale IQ from 45 to approximately 63 after an average of 16 years of treatment, while individuals with moderate phenotypes achieved near-borderline intellectual functioning. In addition to cognitive gains, patients experienced progressive amelioration of motor and autonomic symptoms: spasticity, truncal hypotonia, dysphagia, hypersalivation, and oculogyric crises were markedly reduced within 6 to 12 months of treatment, and seizure frequency decreased substantially. Other systemic features, such as feeding difficulties, recurrent pneumonia, hyperthermia, and abnormal odor, also improved or resolved (165). Despite early diagnosis and continuous treatment with sapropterin and neurotransmitter precursors, several individuals exhibited subtle residual neurological and psychiatric manifestations (154, 159, 168) including seizures, mild spasticity, dystonia, clumsiness, fatigability, anxiety, and mood or sleep disturbances, as well as fine motor incoordination, tremor, perioral tremor, and occasional motor or facial tics (156). In the cohort reported by Manti et al., neurological impairments of varying severity were observed in more than half of the 28 patients examined, including individuals diagnosed both through newborn screening and later in life. These manifestations were markedly more frequent among patients with a severe metabolic phenotype (72%) than among those with a mild phenotype (about one third). Movement disorders were the most commonly reported neurological features, affecting close to half of the cohort, and encompassed bradykinesia, chorea, athetosis, dystonia, tics, ataxia, clumsiness, and arm tremor. Milder movement abnormalities, such as clumsiness, tics, and mild generalized chorea, were predominantly observed in patients with a mild metabolic phenotype. Autonomic dysfunction (including ptosis, hypersalivation, sweating, and temperature instability) was reported in approximately one third of patients and was confined to those with a severe phenotype. Sleep disorders were also relatively frequent (2 patients with mild metabolic phenotype and 7 with the severe form), whereas epilepsy (3 patients) and scoliosis (4 patients) were less commonly described. Intellectual disability was identified in nearly one third of patients and was exclusively observed in individuals with a severe biochemical phenotype, with a higher frequency among those treated later (about half) compared with those treated early (about one third) (127).

Concerning cognitive outcomes in patients with early treatment, normal or borderline IQ was reported when therapy was initiated, respectively, within the first or second month of life (171, 174, 175); other studies described delayed development in early childhood with IQs generally reaching the normal range by school age, followed by a mild decline in older individuals (156); other cohorts showed persistent neurodevelopmental impairment despite early intervention (159, 176, 177). Some patients with mild forms of PTPSd, diagnosed early, remained neurologically and cognitively stable even after discontinuing treatment (171) or under only BH4 supplementation (178).

Recognizing the importance of dopamine and serotonin for the development of EF and for adaptive behavior, several papers employed standardized tools to explore both (117, 127) and psychiatric issues in young adult patients with PTPSd (127). Severe impairment of EF tasks was detected in three individuals whose treatment onset was delayed by 2.5, 27, and 41 years, respectively (117). In another study, 8 of the 20 patients assessed for EF scored above the clinical cut-off on at least one BRIEF subscale and 7 of the 12 evaluated showed significant impairment in adaptive behavior on the VABS-II (127). Moreover, 12 out of 28 patients met the criteria for a psychiatric diagnosis, including generalized anxiety disorder in half of them, depressive disorder in 4, obsessive-compulsive disorder in 2, and attention deficit hyperactivity disorder (127). The study by Schuler et al. examines eight patients with PTPSd, in whom traditional treatment with BH4, a phe-restricted diet, levodopa/carbidopa, and 5-hydroxytryptophan often leads to fluctuating motor and cognitive outcomes, particularly at higher levodopa doses. The authors report that adding the selective MAO-B inhibitor selegiline significantly improved clinical status, reduced levodopa requirements by approximately 40%, and in some cases allowed a reduction in 5-hydroxytryptophan dosage. Symptoms such as convulsions and levodopa–related on–off phenomena resolved, and patients demonstrated better behavior and overall performance. Notably, the patient who began selegiline during the first week of life achieved normal motor and cognitive development (179).

Concerning SRd, global developmental delay leading to intellectual disability, bulbar dysfunction, hypo- and hyperkinetic movement disorders with diurnal fluctuations, dysarthria, and ataxia in some patients, along with sleep disruption, are the most frequent symptoms of the disease before treatment (90, 115, 180–183). Symptoms typically begin in infancy, with a mean age of onset of about 7 months, but the diagnosis is often considerably delayed (115, 183). Despite marked and sustained motor and bulbar improvement following levodopa administration, moderate-to-severe cognitive difficulties often persist (154, 180–182, 184, 185), although in some cases clinical benefit has been reported only with BH4 supplementation rather than with levodopa (90). This suggests that dopaminergic replacement effectively restores motor function but does not fully reverse the underlying neurodevelopmental deficit. However, cognitive improvement was reported in 34% of patients (115), and some later-treated individuals in some studies (186, 187) even achieved normal schooling, suggesting that additional factors—possibly genetic or environmental—may modulate the cognitive trajectory in SRd. Early treatment of this condition is compatible with a complete resolution of neurological and neurodevelopmental disorders (174, 181, 188).

A milder phenotype has recently been reported in five subjects from the same pedigree harboring heterozygous variants in both *SPR* and *DHFR*. The disease presented in childhood or puberty with lower limb exercise dystonia, progressing to dystonia-parkinsonism with pyramidal signs in some patients. The cumulative effect of the partial loss of function of the two enzymes has been suggested to cause this condition (189).

DHPRd is likely the most severe disorder among BH4 deficiencies, being associated with poor clinical outcome and a high burden of neurological sequelae in cases of delayed diagnosis (126, 142), while residual neurological impairment is also reported in an unexpected high percentage of patients treated early (2, 111, 142, 168, 176, 190–192). Systematic long-term follow-up studies of the outcome of patients with DHPRd are lacking. Jäggi et al. reported that only 2 out of 10 patients, aged 3 and 24 years, who were treated at the age of 1 month and 3 weeks, respectively, did not suffer from intellectual disability. All the others were mildly to severely disabled (including a patient treated as early as 3 weeks), with neurological impairment and movement disorders (8 out of 10) and severe epilepsy (6 out of 10) (123). Epilepsy may be an early symptom, but it may also occur later in childhood despite strict treatment (123–125, 144, 161). Similarly, in an Irish cohort with DHPRd, despite early initiation of therapy, neurological comorbidities were still observed in two adult patients, including mild to moderate intellectual disability, language delay, intermittent tremor, and mild pyramidal signs in the lower limbs, as well as a single seizure in one case. In contrast, the two youngest patients, aged 17 months and 9 months at their last follow-up, showed normal psychomotor development and no neurological abnormalities (193). More recent series, while confirming the variability in clinical outcomes in early-treated subjects and the lack of predictive biomarkers, support the impact of early monoaminergic supplementation in preventing permanent neurological disability, particularly regarding cognitive development and neuropsychological functions (154, 194).

Peculiar neurological outcomes were observed in two unrelated patients, aged 7 and 22 years, diagnosed by newborn screening with DHPRd and treated since infancy with standard therapy. Despite optimal management, residual motor and behavioral symptoms, as well as fluctuating hyperprolactinemia, persisted; however, the introduction of the dopamine agonist pramipexole led to marked and sustained clinical and biochemical improvement over a two-year follow-up, without adverse reactions (195). A 9-year-old patient with moderate intellectual disability developed transient episodes of acute parkinsonism and autonomic dysfunction despite starting treatment at 3 months; this patient later experienced severe depression (196). Slow progressive parkinsonism was also reported in a 16-year-old boy with mild intellectual disability, who had been under treatment since the age of 2 months (197). Splicing defects and non-conservative missense mutations, which substantially reduce protein expression or stability, are associated with severe clinical phenotypes of DHPRd and an unfavorable neurological outcome (198).

##### 3.2.6.1 Pregnancy

Giżewska et al. described two pregnancies in a woman diagnosed with PTPSd at age 18 years. Her first pregnancy occurred 6 months later, at 18.5 years. The patient continued treatment with BH4, levodopa/carbidopa, and 5-hydroxytryptophan (5-HTP) throughout gestation. The BH4 dosage was gradually increased to maintain plasma Phe concentrations within the recommended range, while only minor adjustments were made to the doses of neurotransmitter precursors. Despite episodes of threatened preterm labor, gestational diabetes, and vaginitis, the pregnancy resulted in the premature birth of a girl at 31 weeks with intrauterine growth retardation. Neurological examination revealed mild left-sided spastic hemiparesis, and cerebral palsy in the form of spastic hemiplegia was diagnosed; neurodevelopmental rehabilitation was therefore initiated. At 8 years of age, the child showed above-average intellectual performance (IQ = 127) despite residual motor deficits. A second pregnancy occurred two and a half years later. This pregnancy progressed to term and resulted in the birth of a healthy boy with normal psychomotor and cognitive development (IQ = 110) (199).

One adult female with DHPRd experienced three pregnancies: the first resulted in a healthy infant delivered by emergency cesarean section due to fetal distress, the second ended in miscarriage at 14 weeks, and the third resulted in the birth of another healthy child (193).

Finally, a multicenter retrospective study described 16 pregnancies in seven women with different BH4 deficiencies (including GTPCHd, PTPSd, DHPRd, and SRd) who were followed in specialized European metabolic centers participating in the International Working Group on Neurotransmitter Related Disorders (iNTD) registry. Overall, pregnancies were well tolerated, and obstetric outcomes were largely favorable, with one miscarriage and few complications such as prematurity or intrauterine growth restriction, none of which appeared to be related to the standard drug treatment for BH4 deficiencies (110, 200).

### 3.3 Recommendation for future clinical studies

The current treatment for BH4 deficiency was developed based on a noncontrolled clinical trial that identified a new syndromic class of movement disorders (Dopa-Responsive-Dystonia) (5) or on a biochemical background-derived approach (recessive disorders of BH4 metabolism). Therefore, standardized clinical tools to measure treatment effects, as now required for controlled trials, have never been established. For future studies or routine clinical monitoring, Table 3 summarizes standardized tools currently used to assess and score clinical impairment in children and adults with MDs, accounting for both motor and nonmotor symptoms. In children with BH4 deficiencies, assessment of neurodevelopmental impairment using age-specific tests is mandatory, and the possibility that the dosage of effective drugs for MD may be inadequate to prevent or improve cognitive impairment should be considered and addressed in monitoring. Many standardized tools are available to assess cognitive, adaptive, and behavioral problems across childhood, adolescence, and adulthood. Clinical follow-up should be scheduled based on the severity of neurological impairment, the patient’s age, treatment response and adherence, drug adverse effects, and comorbid conditions, including behavioral and psychiatric disorders.

**Table 3:** Standardized tools assessing motor and non-motor symptoms in BH4 deficiencies.

Test/Scale	Target	Age range	Test structure	Comment	Administration time
Motor development/Movement disorder assessment					
Alberta Infant Motor Scale (AIMS).	Maturation of gross motor skills of infants.	0–18 months	Observational scoring tool including 58 items divided into four position-centric sub-scales: Prone (21 items), Supine (9 items), Sitting (12 items), Standing (16 items).	A screening tool to detect and track early developmental delays. Designed to detect alterations in gross motor skill developmental trajectories.	20′–30′
Peabody Developmental Motor Scales-2rd Edition (PDMS-2).	Gross and fine motor assessment of children and developmental progress.	0–5 years	Structured in six subtests (Reflexes, Stationary, Locomotion, Object Manipulation, Grasping, Visual-Motor Integration). The scores protocol has three levels (performed the task correctly: 2 points; performed tasks partially: 1 point; not execute the development criteria correctly: zero points).	Designed to explore gross and fine motor development and follow-up.	40′–60′
Bayley Scales of Infant and Toddler Development-3rd Edition (Bayley-III).	Formal developmental assessment tool for diagnosing developmental delays in early childhood.	16 days to 42 months/15 days.	Items: Cognitive scale, Language (receptive and expressive) scale, Motor scale (fine and the gross motor domains), Social–Emotional Scale.	Designed to detect neurodevelopmental disorders.	30′–90′ according to the patient’s age.
Movement Disorder Childhood Rating Scale 0–3 (MD-CRS 0–3).	Assessment of dyskinesias during infancy and early childhood.	0–3 years	General assessment (Part I), movement-disorder severity (Part II). Part I: motor function, oral/verbal function, and attention/alertness (10 items). Part II: intensity of movement abnormality at rest or during spontaneous behavior.	Designed to assess the severity of movement disorders and their impact on daily life in pediatric patients.	20′–30′
Gross Motor Function Measure-88 (GMFM-88).	Assessment of cerebral palsy (CP).	0–18 years	The GMFM-88 consists of five dimensions: (A) lying and rolling; (B) sitting; (C) crawling and kneeling; (D) standing; (E) walking, running, and jumping. Raw and percent scores are calculated for each of the five dimensions, as well as a total score.	Designed to evaluate and monitor changes in gross motor function, especially in children with CP.	30′–60′ according to the patient’s age.
Movement disorder-childhood rating scale (MD-CRS).	Dystonia and other dyskinesias assessment and follow-up.	4–18 years	General Assessment (Part I) and Movement-Disorder Severity (Part II). Part I: motor function, oral/verbal function, self-care, and attention/alertness (15 items). Part II: severity of the prevalent movement abnormality at rest and during the execution of specific tasks.	Designed for movement disorders in children and adolescents.	20′–30′
Burke-Fahn-Marsden Dystonia Rating Scale (BFMDRS).	Dystonia severity assessment and follow-up.	4–16 years	Consists of a movement [Burke-Fahn-Marsden Movement Scale (BFMMS), and disability Burke-Fahn-Marsden Disability Scale (BFMDS)] subscales. The BFMMS measures dystonia in 9 body regions (including eyes, mouth, speech and swallowing, neck, trunk, arms, and legs). The BFMDS consists of parental- or self-reported daily activities (involving speech, handwriting, feeding, eating, swallowing, hygiene, dressing, and walking).	The most used scale for the assessment of dystonia in children and adolescents. BFMMS scores are age-dependent, at least until 16 years of age.	30′–60′ according to the patient’s condition.
Fahn Tolosa-Marin Tremor Rating Scale (FTMTRS).	Essential tremor and Parkinson’s disease.	>6 years	It is composed of 21 items, organized into 3 parts. The motor section includes assessment of tremor amplitude in 9 specific anatomic locations (part A) as well as during specific upper extremity motor tasks (part B: handwriting, spiral drawing, pouring). The part C includes assessment of tremor and its subject-reported interference with specific activities of daily living.	It is a comprehensive tool designed to assess the severity of tremors across various conditions.	20′–30′
MDS-Unified Parkinson’s Disease Rating Scale (MDS-UPDRS).	Parkinson’s Disease and parkinsonism.	>Late childhood	Includes: Part I (non-motor experiences of daily living), Part II (motor experiences of daily living), Part III (motor examination), Part IV (motor complications).	It is the most adopted scale to assess symptoms and disability due to Parkinson’s Disease and parkinsonism.	30′–60′ according to the patient’s condition.
Neurodevelopmental assessment					
Ages and Stages Questionnaires®, Third Edition.	Developmental delay and follow-up.	1–66 months	Areas: Communication, Gross motor, Fine motor, Problem solving, Personal-social.	Designed to screen young children’s developmental progress, helping to identify children who may need further evaluation or early intervention.	10′–15’
Bayley Scales of Infant and Toddler Development, 3rd Edition (Bayley-III).	Developmental delay and follow-up.	16 days to 42 months/15 days.	Items: Cognitive scale, Language (receptive and expressive) scale, Motor scale (fine and gross motor domains), Social–Emotional Scale.	Designed to score the severity of neurodevelopmental delay.	30′–90′ according to the patient’s age.
Griffiths Scales of Child Development, 3rd Edition (Griffiths-III).	Developmental delay and follow-up.	0–72 months	Provides a detailed profile of general developmental quotient (GDQ) and the assessment of five domains: foundations of learning (subscale A), language and communication (subscale B), eye and hand coordination (subscale C), personal–social–emotional (subscale D), and gross motor (subscale E).	Designed to score the severity of neurodevelopmental delay.	60′–90′ according to the patient’s age.
Cognitive impairment					
Leiter International Performance Scale, Third Edition (Leiter-3).	Cognitive impairment (IQ).	>3 years	Cognitive Battery: assesses problem-solving and reasoning abilities. Attention and Memory Battery: measures working memory and sustained attention.	It is a nonverbal intelligence test designed to evaluate cognitive abilities without relying on verbal language skills.	60′–90′ according to the patient’s age.
Wechsler Preschool and Primary Scale of Intelligence, Fourth Edition (WPPSI-IV).	Cognitive impairment (IQ).	2 years and 6 months −7 years and 7 months.	Includes different subtests grouped into index scores: (1) Verbal Comprehension Index (VCI); (2) Visual–Spatial Index (VSI); (3) Fluid Reasoning Index (FRI); (4) Working Memory Index (WMI); (5) Processing Speed Index (PSI).	Measures Cognitive abilities, verbal and non-verbal reasoning, working memory, and processing speed.	45′–60′
Wechsler Intelligence Scale for Children, Fourth and Fifth Edition (WISC-IV or WISC-V).	Cognitive impairment (IQ).	6–16 years and 11 months.	Consists of four indices: verbal comprehension, perceptual reasoning, working memory, and processing speed.	Idem	60′–90′
Wechsler Adult Intelligence Scale, Fourth Edition (WAIS-IV).	Cognitive impairment (IQ).	≥17 years	Idem	Idem	60′–90′
The Mini Mental State Examination (MMSE).	Cognitive decline.		Includes 11 questions testing five areas of cognitive function: orientation, registration, attention and calculation, recall, and language. The maximum score is 30. A score of 23 or lower is indicative of cognitive impairment.	screening tool for cognitive impairment with older, community-dwelling, hospitalized, and institutionalized adults.	5′–10′ to use repeatedly and routinely.
Trail Making Test Part A and Part B.	Executive function impairment.	>12 years	Trail Making Test A = measures: processing speed, visual scanning, and motor coordination. Trail Making Test B = measures cognitive flexibility, task-switching ability, and working memory. Only the time needed to complete the test is measured	Forms A and B require focused attention for successful performance. In addition, Form B requires the patient to switch cognitive sets between numbers and letters. Both forms are highly dependent upon motoric speed and may not be appropriate for patients with motor impairment.	5′–10′
The semantic fluency test (SFT).	Executive function impairment.	>5 years	Subjects are asked to generate as many words as possible from a given semantic category (e.g., “animals”) within a limited time, usually 1 min.	Measures flexibility and planning.	3′
The phonemic fluency test (PFT).	Executive function impairment.	>5 years	Subjects are asked to orally produce as many words as possible beginning with a specific letter (P, F, and L). The test consists of three trials of 1 min each.	Measures flexibility and planning.	3′
Rey Figure Test with copy (RFTC) and from memory (RFTM).	Executive function impairment.	>4 years	Subjects copy complex geometric shapes and then reproduce them from memory. It includes immediate copy and delayed recall.	These tests involve sustained attention, planning, and visual organization of complex data, as well as visual memory.	10′–20′
The Brief Rating Inventory of Executive Function (BRIEF child, adolescent, and adult version).	Executive function impairment.	>2 years	Measures different aspects of executive function to produce two indexes [Behavior Regulation Index (BRI) and the Metacognition Index (MI)] and one composite summary score (Global Executive Composite, GEC).	Its ecological validity is considered high because it provides insights into how executive functions manifest in daily life.	10′–15′
Adaptive, emotional, and behavioral difficulties					
Vineland Adaptive Behavior Scale Second Edition (VABS-II).	Adaptive behaviors.	0–90 years	Consists of 11 subdomains grouped into four domain composites (Communication, Daily Living Skills, Socialization, and Motor Skills), with the domain composites used to derive the adaptive behavior composite.	Semi-structured interviews and questionnaire that assess personal and social skills.	30′–45′
The Achenbach System of Empirically-Based Assessments (ASEBA).	Emotional and behavioral problems	>1.5 years	A set of standardized questionnaires used to assess behavioral, emotional, social, and thought problems in individuals, as well as their strengths and adaptive functioning.	Self-report assessments.	10′–15′
State–Trait Inventory form Y (STAI-Y).	Anxiety disorders.	>12 years	Is a self-report assessment instrument that permits a distinction between existing anxiety and predisposition to an anxious reaction as a personality characteristic.	Self-report assessments.	20′
Beck Depression Inventory (BDI-II).	Mood disorders.	>13 years	A 21-item self-report instrument that measures the intensity of depressive symptoms. Items are summed to produce a single depression summary score and somatic–affective, and cognitive factors.	Self-report assessments.	5′
Sleep disorders					
The Sleep Disturbance Scale for Children (SDSC).	Sleep disturbances in childhood and adolescence.	Infancy to adolescence.	A 26-item scale including assessments of Disorders of Initiating and Maintaining Sleep, Sleep Breathing Disorders, Disorders of Arousal/Nightmares, Sleep/Wake Transition Disorders, Disorders of Excessive Somnolence, and Sleep Hyperhydrosis (excessive sweating).	Parent-completed rating scale.	10′–15′
Epworth Sleepiness Scale.	Sleep disturbances.	Adulthood and old people.	8 questions concerning the subject’s usual chances of dozing off or falling asleep while engaged in eight different activities.	Self-administered questionnaire.	2′–3′

## 4 Conclusion

BH4 deficiencies cause early and persistent depletion of monoaminergic neurotransmitters in the brain, disrupting neurological and mental functions from birth through adulthood. Outcome studies, which frequently combine early and late-treated subjects and sometimes include participants with differing diagnoses, still report a high percentage of patients experiencing ongoing neurological, cognitive, and behavioral disabilities. The lack of harmonization in assessing the neurological impairments caused by these diseases is a current limit for the comprehensive understanding of many clinical aspects, hampering the personalization of treatment based on individual disability profiles. Open problems concern the long-term outcomes of the diseases, as well as determinants of specific risks for each (etc.) Beyond a few metabolic and instrumental tests required in specific situations, future studies require standardized clinical evaluations of impaired function to optimize treatment and identify outcome predictors.

## Data Availability

This systematic review does not generate or include any datasets; it synthesizes data from published retrospective observational studies via citations and references only, with no shareable raw data or supplementary files containing datasets. Requests for source study data should be directed to the original authors/publications.

## Glossary

AADC: aromatic amino acid decarboxylase
ADC: apparent diffusion coefficient
AD-GTPCHd: autosomal dominant guanosine triphosphate cyclohydrolase I deficiency
AR-GTPCHd: autosomal recessive guanosine triphosphate cyclohydrolase I deficiency
BFMDRS: Burke-Fahn-Marsden Dystonia Rating Scale
BH4: tetrahydrobiopterin
CFD: Central Folate Deficiency
CSF: cerebrospinal fluid
DAT: dopamine transporter
DHPR: q-dihydropyridine reductase
DHPRd: q-dihydropyridine reductase deficiency
DRD: dopa-responsive dystonia
DHFR: dihydrofolate reductase
EEG: electroencephalography
EF: executive functions
FP-CIT SPECT: 123-ioflupane single photon emission computed tomography
GH: growth hormone
GTPCH: guanosine triphosphate cyclohydrolase
5-HIAA: 5-hydroxyindoleacetic acid
HPA: hyperphenylalaninemia
HVA: homovanillic acid
IQ: intellectual quotient
MD: movement disorder
MRI: magnetic resonance imaging
MDS-UPDRS: MDS-Unified Parkinson’s Disease Rating Scale
5-MTHF: 5-methyltetrahydrofolate
PD: Parkinson disease
PDCC: pterin-4-alpha-carbinolamine dehydratase
PDCCd: pterin-4-alpha-carbinolamine dehydratase deficiency
[F-18]FDOPA PET: 18F-Fluorodopa positron emission tomography
Phe: phenylalanine
PKU: phenylketonuria
PTPS: 6-pyruvoyltetrahydropterin synthase
PTPSd: 6-pyruvoyltetrahydropterin synthase deficiency
QOL: quality of life
SN: substantia nigra
SR: sepiapterin reductase
SRd: sepiapterin reductase deficiency
TSH: thyroid-stimulating hormone
