# Supplementary material for: Follow-up and outcome of patients with primary BH4 deficiencies
Source: Front Neurol. 2026 Jul 16;17:1793300. doi: 10.3389/fneur.2026.1793300 (PMC13422167; doi:10.3389/fneur.2026.1793300)
Supplement: Supplementary file 1 [file Table_1.DOCX]

**Supplementary Table 1**

Adapted version of the Newcastle-Ottawa scale used in the present study for quality assessment (maximum 9 stars).

| **Selection (maximum 4 stars):** |
| --- |
| ***Is the case definition adequate?***  a) Yes, with standard diagnostic criteria (i.e., DSM, ICD, standardized diagnostic tools).  b) Based on parental/self-reports or on clinical interviews.  c) No description.  ***Representativeness of the cases:***  a) Consecutive or obviously representative series of cases.* b) Potential for selection biases or not stated.  ***Selection of controls:***  a) Community controls. *  b) Hospital controls or parents/siblings.  c) No description or absence of controls.  ***Definition of controls:***  a) No history of disease (healthy controls). *  b) Other diagnoses.  c) No description or absence of controls. |
| **Comparability (maximum 2 stars):** |
| ***Comparability of cases and controls based on the design or analysis***  a) Study controls for age and sex. * b) Study controls for IQ. *  c) Matching only for age or sex, or no matching neither for age and sex nor for IQ |
| **Methods and procedure (maximum 3 stars):** |
| ***Experimental procedure:***  a) The experimental procedure is well described and includes valid measures and methods. *  b) Poor description or no description of the procedure.  ***Same experimental procedure for cases and controls:***  a) Yes. *  b) No.  c) No description or absence of controls  ***Drop-out rate:***  a) No drop-outs or same rate for all groups. *  b) Different rate or not defined  c) No description or absence of more than one group |

Supplementary Table 1. Details on quality assessment indices for the retrieved studies

| **Study** | **Selection** | | | | **Comparability** | **Methods and procedure** | | | **Total** |
| --- | --- | --- | --- | --- | --- | --- | --- | --- | --- |
|  | **Is the case definition adequate?** | **Representativeness of the cases:** | **Selection of controls:** | **Definition of controls:** | **Comparability of cases and controls based on the design or analysis** | **Experimental procedure:** | **Same experimental procedure for cases and controls:** | **Drop-outs rate** |  |
| Almannai et al., 2019 | * (a) | * (a) | (c) | (c) | (c) | * (a) | *(a) | * (a) | 5 |
| Brique et al., 1999 | * (a) | *(a) | (c) | (c) | (c) | * (a) | * (a) | * (a) | 5 |
| Tachi et al., 2011 | * (a) | *(a) | (c) | (c) | (c) | * (a) | * (a) | * (a) | 5 |
| Bernal-Pacheco et al., 2012 | * (a) | *(a) | (c) | (c) | (c) | * (a) | * (a) | * (a) | 5 |
| Hu et al., 2010 | * (a) | *(a) | (c) | (c) | (c) | * (a) | * (a) | * (a) | 5 |
| Yang et al., 2018a | * (a) | *(a) | (c) | (c) | (c) | * (a) | * (a) | * (a) | 5 |
| Kang et al., 2004 | * (a) | *(a) | *(a) | *(a) | (c) | * (a) | * (a) | * (a) | 7 |
| Markova et al., 1999 | * (a) | *(a) | (c) | (c) | (c) | * (a) | * (a) | * (a) | 5 |
| Hong et al., 2001 | * (a) | *(a) | (c) | (c) | (c) | * (a) | * (a) | * (a) | 5 |
| Smooker et al., 1999 | * (a) | *(a) | (c) | (c) | (c) | * (a) | * (a) | * (a) | 5 |
| Skrygan et al., 2001 | * (a) | *(a) | (c) | (c) | (c) | * (a) | * (a) | * (a) | 5 |
| Terao et al., 2024 | * (a) | *(a) | *(a) | *(a) | *(a) | * (a) | * (a) | * (a) | 8 |
| Opladen et al., 2012 | * (a) | *(a) | (c) | (c) | (c) | * (a) | * (a) | * (a) | 5 |
| Ling et al., 2011 | * (a) | *(a) | (c) | (c) | (c) | * (a) | * (a) | * (a) | 5 |
| Keller et al., 2021 | * (a) | *(a) | *(a) | *(a) | *(b) | * (a) | * (a) | * (a) | 8 |
| Wassenberg et al., 2020 | * (a) | *(a) | (c) | (c) | (c) | * (a) | * (a) | * (a) | 5 |
| Novelli et al., 2024 | * (a) | *(a) | *(a) | *(a) | (c) | * (a) | * (a) | * (a) | 7 |
| Trender-Gerhard et al., 2009 | * (a) | *(a) | *(a) | *(a) | (c) | * (a) | * (a) | * (a) | 7 |
| De Souza et al., 2017 | * (a) | *(a) | (c) | (c) | (c) | * (a) | * (a) | * (a) | 5 |
| Kuseyri Hübschmann et al., 2021 | * (a) | *(a) | *(a) | *(a) | *(a) | * (a) | * (a) | * (a) | 8 |
| Kostić et al., 2020 | * (a) | *(a) | *(a) | *(a) | (c) | * (a) | * (a) | * (a) | 7 |
| Talvik et al., 2010 | * (a) | *(a) | (c) | (c) | (c) | * (a) | * (a) | * (a) | 5 |
| Chen et al., 2020 | * (a) | *(a) | (c) | (c) | (c) | * (a) | * (a) | * (a) | 5 |
| Tamaru et al., 1998 | * (a) | *(a) | (c) | (c) | (c) | * (a) | * (a) | * (a) | 5 |
| De Castro-Hamoy et al., 2025 | * (a) | *(a) | (c) | (c) | (c) | * (a) | * (a) | * (a) | 5 |
| Rudakou et al., 2019 | * (a) | *(a) | (c) | (c) | (c) | * (a) | * (a) | * (a) | 5 |
| Ye et al., 2012 | * (a) | *(a) | *(a) | *(a) | *(a) | * (a) | * (a) | * (a) | 8 |
| Schuler et al., 2000 | * (a) | *(a) | *(a) | *(a) | *(a) | * (a) | * (a) | * (a) | 8 |
| Han et al., 2015 | * (a) | *(a) | *(a) | *(a) | *(a) | * (a) | * (a) | * (a) | 8 |
| Ray et al., 2021 | * (a) | *(a) | *(a) | *(a) | *(a) | * (a) | * (a) | * (a) | 8 |
| Porta et al., 2009 | * (a) | *(a) | (c) | (c) | (c) | * (a) | * (a) | * (a) | 5 |
| Zhang et al., 2017 | * (a) | *(a) | (c) | (c) | (c) | * (a) | * (a) | * (a) | 5 |
| Yang et al., 2018b | * (a) | *(a) | (c) | (c) | (c) | * (a) | * (a) | * (a) | 5 |
| Steinberger et al., 2000 | * (a) | *(a) | (c) | (c) | (c) | * (a) | * (a) | * (a) | 5 |
| Tanaka et al., 2007 | * (a) | *(a) | *(a) | *(a) | *(b) | * (a) | * (a) | * (a) | 8 |
| Clot et al., 2009 | * (a) | *(a) | (c) | (c) | (c) | * (a) | * (a) | * (a) | 5 |
| Van Hove et al., 2005 | * (a) | *(a) | *(a) | *(a) | *(a) | * (a) | * (a) | * (a) | 8 |
| Karam et al., 2010 | * (a) | *(a) | (c) | (c) | (c) | * (a) | * (a) | * (a) | 5 |
| Irons et al., 1987 | * (a) | *(a) | (c) | (c) | (c) | * (a) | * (a) | * (a) | 5 |
| Cao et al., 2010 | * (a) | *(a) | (c) | (c) | (c) | * (a) | * (a) | * (a) | 5 |
| Yoshino et al., 2028 | * (a) | *(a) | (c) | (c) | (c) | * (a) | * (a) | * (a) | 5 |
| Varghaei et al., 2021 | * (a) | *(a) | (c) | (c) | (c) | * (a) | * (a) | * (a) | 5 |
| Fernandenz Ramos et al., 2022 | * (a) | *(a) | (c) | (c) | (c) | * (a) | * (a) | * (a) | 5 |
| Robinson et al., 1999 | * (a) | *(a) | (c) | (c) | (c) | * (a) | * (a) | * (a) | 5 |
| Garavaglia et al., 2004 | * (a) | *(a) | (c) | (c) | (c) | * (a) | * (a) | * (a) | 5 |
| Wu-Chou et al., 2019 | * (a) | *(a) | (c) | (c) | (c) | * (a) | * (a) | * (a) | 5 |
| Steinberger et al., 1998 | * (a) | *(a) | (c) | (c) | (c) | * (a) | * (a) | * (a) | 5 |
| Coskun et al., 1993 | * (a) | *(a) | (c) | (c) | (c) | * (a) | * (a) | * (a) | 5 |
| Li et al., 2022 | * (a) | *(a) | (c) | (c) | (c) | * (a) | * (a) | * (a) | 5 |
| Pinto et al., 2021 | * (a) | *(a) | (c) | (c) | (c) | * (a) | * (a) | * (a) | 5 |
| Hanajima et al., 2007 | * (a) | *(a) | (c) | (c) | (c) | * (a) | * (a) | * (a) | 5 |
| Tassin et al., 2000 | * (a) | *(a) | (c) | (c) | (c) | * (a) | * (a) | * (a) | 5 |
| Manti et al., 2020 | * (a) | *(a) | *(a) | *(a) | *(b) | * (a) | * (a) | * (a) | 8 |
| Lee et al., 2006 | * (a) | *(a) | *(a) | *(a) | *(a) | * (a) | * (a) | * (a) | 8 |
| Liu et al., 2008 | * (a) | *(a) | *(a) | *(a) | *(a) | * (a) | * (a) | * (a) | 8 |
| Wang et al., 2006 | * (a) | *(a) | *(a) | *(a) | *(a) | * (a) | * (a) | * (a) | 8 |
| Porta et al., 2016a | * (a) | *(a) | *(a) | *(a) | (c) | * (a) | * (a) | * (a) | 7 |
| Chaiyasap et al., 2017 | * (a) | *(a) | *(a) | *(a) | (c) | * (a) | * (a) | * (a) | 7 |
| Wu et al., 2008 | * (a) | *(a) | (c) | (c) | (c) | * (a) | * (a) | * (a) | 5 |
| Dudesek et al., 2001 | * (a) | *(a) | (c) | (c) | (c) | * (a) | * (a) | * (a) | 5 |
| Romstad et al., 2000 | * (a) | *(a) | (c) | (c) | (c) | * (a) | * (a) | * (a) | 5 |
| Foroozani et al., 2015 | * (a) | *(a) | (c) | (c) | (c) | * (a) | * (a) | * (a) | 5 |
| Carducci et al., 2020 | * (a) | *(a) | (c) | (c) | (c) | * (a) | * (a) | * (a) | 5 |
| Ferrè et al., 2013 | * (a) | *(a) | (c) | (c) | (c) | * (a) | * (a) | * (a) | 5 |
| Thony et al., 1998 | * (a) | *(a) | (c) | (c) | (c) | * (a) | * (a) | * (a) | 5 |
| Hahn et al., 2001 | * (a) | *(a) | (c) | (c) | (c) | * (a) | * (a) | * (a) | 5 |
| Passaretti et al., 2022 | * (a) | *(a) | (c) | (c) | (c) | * (a) | * (a) | * (a) | 5 |
| Lopez-Laso et al., 2011 | * (a) | *(a) | *(a) | *(a) | *(b) | * (a) | * (a) | * (a) | 8 |
| Camargos et al., 2008 | * (a) | *(a) | (c) | (c) | (c) | * (a) | * (a) | * (a) | 5 |
| Lee et al., 2013 | * (a) | *(a) | (c) | (c) | (c) | * (a) | * (a) | * (a) | 5 |
| Ohta et al., 2006 | * (a) | *(a) | (c) | (c) | (c) | * (a) | * (a) | * (a) | 5 |
| Bardien et al., 2010 | * (a) | *(a) | (c) | (c) | (c) | * (a) | * (a) | * (a) | 5 |
| Naiya et al., 2012 | * (a) | *(a) | (c) | (c) | (c) | * (a) | * (a) | * (a) | 5 |
| Jaggi et al., 2008 | * (a) | *(a) | (c) | (c) | *(b) | * (a) | * (a) | * (a) | 6 |
| Cordeiro et al., 2018 | * (a) | *(a) | (c) | (c) | (c) | * (a) | * (a) | * (a) | 5 |
| Mencacci et al., 2014 | * (a) | *(a) | (c) | (c) | (c) | * (a) | * (a) | * (a) | 5 |
| Li et al., 2025 | * (a) | *(a) | (c) | (c) | (c) | * (a) | * (a) | * (a) | 5 |
| Niederwieser et al., 1987 | * (a) | *(a) | (c) | (c) | (c) | * (a) | * (a) | * (a) | 5 |
| Grimes et al., 2002 | * (a) | *(a) | (c) | (c) | (c) | * (a) | * (a) | * (a) | 5 |
| Kaindl et al., 2005 | * (a) | *(a) | (c) | (c) | (c) | * (a) | * (a) | * (a) | 5 |
| Leuzzi et al., 2010 | * (a) | *(a) | (c) | (c) | *(b) | * (a) | * (a) | * (a) | 6 |
| Kimura et al., 2016 | * (a) | *(a) | (c) | (c) | (c) | * (a) | * (a) | * (a) | 5 |
| Lu et al., 2014 | * (a) | *(a) | (c) | (c) | (c) | * (a) | * (a) | * (a) | 5 |
| Ahn et al., 2019 | * (a) | *(a) | (c) | (c) | (c) | * (a) | * (a) | * (a) | 5 |
| Manzoni et al., 2020 | * (a) | *(a) | (c) | (c) | (c) | * (a) | * (a) | * (a) | 5 |
| Lopez-Laso et al., 2009 | * (a) | *(a) | (c) | (c) | (c) | * (a) | * (a) | * (a) | 5 |
| Neville et al., 2005 | * (a) | *(a) | (c) | (c) | (c) | * (a) | * (a) | * (a) | 5 |
| Friedman et al., 2012 | * (a) | *(a) | (c) | (c) | (c) | * (a) | * (a) | * (a) | 5 |
| Alsubhi et al., 2017 | * (a) | *(a) | (c) | (c) | (c) | * (a) | * (a) | * (a) | 5 |
| Timmers et al., 2022 | * (a) | *(a) | (c) | (c) | (c) | * (a) | * (a) | * (a) | 5 |
| Concolino et al., 2008 | * (a) | *(a) | (c) | (c) | *(b) | * (a) | * (a) | * (a) | 6 |
| Porta et al., 2015 | * (a) | *(a) | (c) | (c) | (c) | * (a) | * (a) | * (a) | 5 |
| Hyodo et al., 2022 | * (a) | *(a) | (c) | (c) | (c) | * (a) | * (a) | * (a) | 5 |
| Wider et al., 2008 | * (a) | *(a) | (c) | (c) | (c) | * (a) | * (a) | * (a) | 5 |
| Porta et al., 2016b | * (a) | *(a) | (c) | (c) | (c) | * (a) | * (a) | * (a) | 5 |
| Bozaci et al., 2021 | * (a) | *(a) | (c) | (c) | (c) | * (a) | * (a) | * (a) | 5 |
| Khani et al., 2021 | * (a) | *(a) | (c) | (c) | (c) | * (a) | * (a) | * (a) | 5 |
| Narahara et al., 2024 | * (a) | *(a) | (c) | (c) | (c) | * (a) | * (a) | * (a) | 5 |
| Gowda et al., 2025 | * (a) | *(a) | (c) | (c) | (c) | * (a) | * (a) | * (a) | 5 |
| Chien et al., 2001 | * (a) | *(a) | (c) | (c) | *(b) | * (a) | * (a) | * (a) | 6 |
| Carducci et al., 2015 | * (a) | *(a) | (c) | (c) | (c) | * (a) | * (a) | * (a) | 5 |
| Lin et al., 2017 | * (a) | *(a) | (c) | (c) | (c) | * (a) | * (a) | * (a) | 5 |
| Uncini et al., 2004 | * (a) | *(a) | (c) | (c) | (c) | * (a) | * (a) | * (a) | 5 |
| Svetel et al., 2017 | * (a) | *(a) | (c) | (c) | (c) | * (a) | * (a) | * (a) | 5 |
| Hsu et al., 2023 | * (a) | *(a) | (c) | (c) | (c) | * (a) | * (a) | * (a) | 5 |
| Weissbach et al., 2021 | * (a) | *(a) | (c) | (c) | (c) | * (a) | * (a) | * (a) | 5 |
| Kuseyri Hübschmann et al., 2018 | * (a) | *(a) | (c) | (c) | (c) | * (a) | * (a) | * (a) | 5 |
| Leuzzi et al., 2012 | * (a) | *(a) | (c) | (c) | *(b) | * (a) | * (a) | * (a) | 6 |
| Alves Júnior et al., 2022 | * (a) | *(a) | (c) | (c) | (c) | * (a) | * (a) | * (a) | 5 |
| Arrabal et al., 2011 | * (a) | *(a) | (c) | (c) | (c) | * (a) | * (a) | * (a) | 5 |
| Chien et al., 2002 | * (a) | *(a) | (c) | (c) | (c) | * (a) | * (a) | * (a) | 5 |
| de Alencar Guerra et al., 2025 | * (a) | *(a) | (c) | (c) | (c) | * (a) | * (a) | * (a) | 5 |
| de la Fuente-Fernández, 1997 | * (a) | *(a) | (c) | (c) | (c) | * (a) | * (a) | * (a) | 5 |
| Hagenah et al., 2005 | * (a) | *(a) | (c) | (c) | (c) | * (a) | * (a) | * (a) | 5 |
| Jarra et al., 2025 | * (a) | *(a) | (c) | (c) | (c) | * (a) | * (a) | * (a) | 5 |
| Jeon et al., 1998 | * (a) | *(a) | (c) | (c) | (c) | * (a) | * (a) | * (a) | 5 |
| Liu et al., 2010 | * (a) | *(a) | (c) | (c) | (c) | * (a) | * (a) | * (a) | 5 |
| Parfyonov et al., 2022 | * (a) | *(a) | (c) | (c) | (c) | * (a) | * (a) | * (a) | 5 |
| Yildiz et al., 2023 | * (a) | *(a) | (c) | (c) | (c) | * (a) | * (a) | * (a) | 5 |
| Shintaku et al., 2013 | * (a) | *(a) | (c) | (c) | (c) | * (a) | * (a) | * (a) | 5 |
| Vela-Amieva et al., 2022 | * (a) | *(a) | (c) | (c) | (c) | * (a) | * (a) | * (a) | 5 |
| Lewthwaite et al., 2015 | * (a) | *(a) | (c) | (c) | (c) | * (a) | * (a) | * (a) | 5 |
| Simaite et al., 2014 | * (a) | *(a) | (c) | (c) | (c) | * (a) | * (a) | * (a) | 5 |
| Timmers et al., 2017 | * (a) | *(a) | *(a) | *(a) | *(a) | * (a) | * (a) | * (a) | 8 |
| Ichinose et al., 1994 | * (a) | *(a) | (c) | (c) | (c) | * (a) | * (a) | * (a) | 5 |
| Miladi et al., 1998 | * (a) | *(a) | (c) | (c) | (c) | * (a) | * (a) | * (a) | 5 |
| Hong et al., 2015 | * (a) | *(a) | (c) | (c) | (c) | * (a) | * (a) | * (a) | 5 |
| Howze et al., 2016 | * (a) | *(a) | (c) | (c) | (c) | * (a) | * (a) | * (a) | 5 |
| ***Note:* the specific fulfilled criterion for the assignment or non-assignment of each star is specified in brackets.** | | | | | | | | | |
